# Supplementary material for: Stacking Model‐Based Classifiers for Dealing With Multiple Sets of Noisy Labels
Source: Biom J. 2025 Mar 12;67(2):e70042. doi: 10.1002/bimj.70042 (PMC11898607; doi:10.1002/bimj.70042)
Supplement: Supplementary file 1 — Supporting Information [file BIMJ-67-e70042-s001.zip › density-based-ensemble-model-main/competitors_real_data/netlab-master/netlab-master/help/dist2.htm]

Netlab Reference Manual dist2


# dist2

## Purpose

Calculates squared distance between two sets of points.

## Synopsis

```
d = dist2(x, c)
```

## Description

`d = dist2(x, c)` takes two matrices of vectors and calculates the
squared Euclidean distance between them. Both matrices must be of the
same column dimension. If `x` has `m` rows and `n` columns, and
`c` has `l` rows and `n` columns, then the result has
`m` rows and `l` columns. The `i, j`th entry is the
squared distance from the `i`th row of `x` to the `j`th
row of `c`.

## Example

The following code is used in `rbffwd` to calculate the activation of
a thin plate spline function.

```
n2 = dist2(x, c);
z = log(n2.^(n2.^2));
```

## See Also

`gmmactiv`, `kmeans`, `rbffwd`

---

**Pages:**
Index


---

Copyright (c) Ian T Nabney (1996-9)
